# Bioretrosynthesis of Functionalized *N*‐Heterocycles from Glucose via One‐Pot Tandem Collaborations of Designed Microbes

**DOI:** 10.1002/advs.202001188

**Published:** 2020-07-21

**Authors:** Jing Feng, Ruifeng Li, Shasha Zhang, Yifan Bu, Yanchun Chen, Yinglu Cui, Baixue Lin, Yihua Chen, Yong Tao, Bian Wu

**Affiliations:** ^1^ CAS Key Laboratory of Microbial Physiological and Metabolic Engineering State Key Laboratory of Microbial Resources Institute of Microbiology Chinese Academy of Sciences Beijing 100101 P. R. China; ^2^ University of Chinese Academy of Sciences Beijing China

**Keywords:** bioretrosynthesis, cascade reactions, multistrain microbial systems, *N*‐heterocycles, synthetic biology

## Abstract

The design of multistrain systems has markedly expanded the prospects of using long biosynthetic pathways to produce natural compounds. However, the cooperative use of artificially engineered microbes to synthesize xenobiotic chemicals from renewable carbohydrates is still in its infancy. Here, a microbial system is developed for the production of high‐added‐value *N*‐heterocycles directly from glucose. Based on a retrosynthetic analysis, eleven genes are selected, systematically modulated, and overexpressed in three *Escherichia coli* strains to construct an artificial pathway to produce 5‐methyl‐2‐pyrazinecarboxylic acid, a key intermediate in the production of the important pharmaceuticals Glipizide and Acipimox. Via one‐pot tandem collaborations, the designed microbes remarkably realize high‐level production of 5‐methyl‐2‐pyrazinecarboxylic acid (6.2 ± 0.1 g L^−1^) and its precursor 2,5‐dimethylpyrazine (7.9 ± 0.7 g L^−1^). This study is the first application of cooperative microbes for the total biosynthesis of functionalized *N*‐heterocycles and provides new insight into integrating bioretrosynthetic principles with synthetic biology to perform complex syntheses.

## Graphical abstract

A microbial system with three engineered *Escherichia coli* strains is developed for the production of *N*‐heterocycles directly from glucose. Via one‐pot tandem collaborations, it realizes high‐level production of 5‐methyl‐2‐pyrazinecarboxylic acid, a key intermediate for the important pharmaceuticals Glipizide and Acipimox. This work provides new insight into integrating bioretrosynthetic principles with synthetic biology to perform complex syntheses.

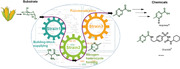

## 1 Introduction

In nature, the coexistence and interaction of microorganisms to accomplish chemically complex tasks^[^ ^1^ ^]^ has significant practical relevance in various fields,^[^ ^2^ ^],^ e.g., bioremediation, food processing, and agriculture. In these complex biochemical processes, sequential inoculation of different microbes provides facile spatial and temporal control of the population composition and metabolic flux and has been used to refine the aromatic composition and sensory properties of wine,^[^ ^3^ ^]^ to facilitate protein hydrolysis for sauce flavorings,^[^ ^4^ ^]^ or to enhance pollutant degradation.^[^ ^5^ ^]^ However, sequential fermentation is rarely applied to the biosynthesis of specific high‐value compounds in chemical industries. An exception is the biotechnological production route of vitamin C (l‐ascorbic acid) developed at our institute.^[^ ^6^ ^]^ In this process, d‐sorbitol is initially oxidized to l‐sorbose by *Gluconobacter oxydans*, followed by a second fermentation step in which *Ketogulonicigenium vulgare* and its helper strain *Bacillus megaterium* mediate the oxidation of l‐sorbose to 2‐keto‐l‐gulonic acid, which is eventually converted by an acid‐catalyzed reaction into l‐ascorbic acid. Dividing the entire process into sequential steps distributes the redox burden of the consecutive oxidations among different species and prevents the undesirable oxidation of d‐sorbitol to glucose and other metabolites mediated by *K. vulgare*. This in tandem operation of multiple microbes provides a clear cost benefit and is currently implemented at the industrial scale in the production of over 150 000 tons of Vitamin C annually. Inspired by the design philosophy of this classic multispecies biotransformation, we envisage that the principles of division of labor and in tandem operation could provide viable solutions for the multistep biosynthesis of valuable xenobiotic compounds that are difficult to assemble or control precisely using a single strain.

Therefore, we explored the biotransformation of renewable biomass into high‐value‐added nitrogen‐containing heterocycles by designed microbes. *N*‐heterocyclic motifs are frequent feedstocks for the production of polymers, pharmaceutical compounds, agrochemicals, etc.^[^ ^7^ ^]^ In particular, 2,5‐dimethylpyrazine (DMP) imparts nutty and toasty tonalities to foods and therefore is widely used as a flavoring additive and odorant in the food industry,^[^ ^8^ ^]^ including as an important flavoring compound in cocoa‐bean‐ or soybean‐based fermented foods. Furthermore, DMP is the precursor for the synthesis of 5‐methyl‐2‐pyrazinecarboxylic acid (MPCA), which is a key component of widely used pharmaceuticals, including Glipizide^[^ ^9^ ^]^ (an antidiabetic medication with annual prescriptions of 17 million in the United States and 30 million in China) and Acipimox^[^ ^10^ ^]^ (a lipid‐lowering drug with annual prescriptions of 5 million in China). Currently, DMP can be synthesized via various routes, including the intramolecular cyclization of 2‐amino‐1‐propanol,^[^ ^11^ ^]^ intermolecular dehydration and dehydrogenation of 1,2‐propanediol and 1,2‐diaminopropane,^[^ ^12^ ^]^ and intermolecular condensation between 1,2‐diaminopropane and methylglyoxal.^[^ ^13^ ^]^ Petroleum‐derived DMP can be subsequently used to synthesize MPCA through a series of oxidation reactions. Studies have been carried out on electrochemical oxidation^[^ ^14^ ^]^ and microbial oxidation,^[^ ^15^ ^]^ but potassium permanganate is effectively used as an oxidation agent^[^ ^16^ ^]^ in the synthetic route for most commercially produced MPCA worldwide. There are, however, several drawbacks associated with these petroleum‐based routes, such as high energy requirements to maintain a high reaction temperature, low product selectivity, and potential environmental hazards from toxic and erosive oxidants. Unlike chemical processes, biotransformation processes are environmentally friendly: for example, reactions occur under mild conditions in the absence of toxic chemicals and metal catalysts, and there is a sustainable supply of raw materials.^[^ ^17^ ^]^ These evident benefits make the production of DMP and MPCA from inexpensive bio‐based carbon sources highly desirable.

**Figure 1:**
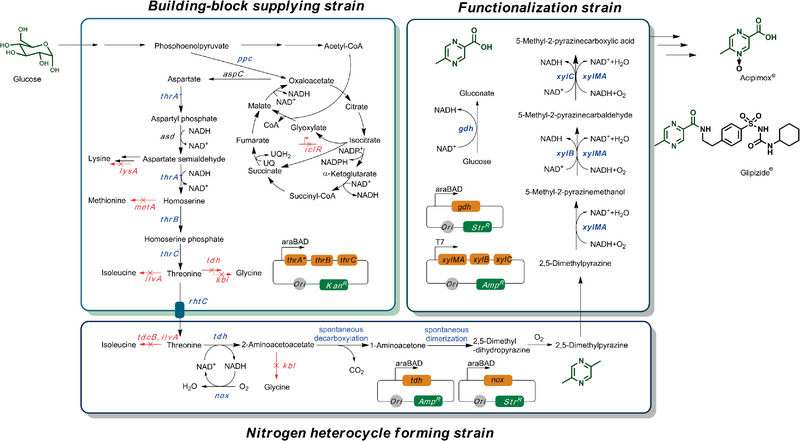
In tandem operation of designed microbes in biotransformation pathway from glucose to *N*‐heterocycles. The arrows indicate metabolic flux from glucose to MPCA; genes marked in blue indicate targets for improvement or introduction, and genes marked in red indicate targets for deletion; *ppc*: phosphoenolpyruvate carboxylase; *iclR*: regulator of glyoxylate operon; thrA*, aspartate kinase; *thrB*, homoserine kinase; *thrC*, threonine synthase; *lysA*: diaminopimelate decarboxylase; *metA*: homoserine succinyltransferase; *tdcB* and *ilvA*: threonine dehydratase; *tdh*: threonine dehydrogenase; *kbl*: 2‐amino‐3‐ketobutyrate coenzyme A (CoA) ligase; *rhtC*: threonine transporter; *xylMA*: xylene monooxygenase; *xylB*: benzyl alcohol dehydrogenase; *xylC*: benzaldehyde dehydrogenase; *nox*: NADH oxidase; *gdh*: glucose dehydrogenase.

However, it is challenging to develop a functional artificial pathway to bridge monosaccharide and non‐natural pharmaceutical compounds, while providing reasonable product yields and titres: design routes invariably require multiple heterologous functionalities, the overexpression of a large number of biosynthetic genes can consume excessive cellular resources, and there is potential interference between different portions of the pathway. As conventional single‐cell engineering cannot easily surmount these technical barriers, we rationally designed a microbial system to accomplish the desired complex tasks. A bioretrosynthetic analysis^[^ ^18^ ^]^ was used to modularize the required biological functions using three cells of the same species: a strain for supplying building blocks, a strain to form *N*‐heterocycles, and a strain for functionalization (**Figure** **1**). The biotransformation was spatially and temporally tuned by sequential inoculations of the functional cells at different stages. Using a one‐pot in tandem scheme, the synthetic microbes directly converted glucose to DMP or MPCA, reaching concentrations up to high levels of 7.9 ± 0.7 and 6.2 ± 0.1 g L^−1^, respectively.

## 2 Results

### 2.1 Retrosynthetic Design of Glucose‐to‐MPCA Biotransformation Pathway

**Figure 2:**
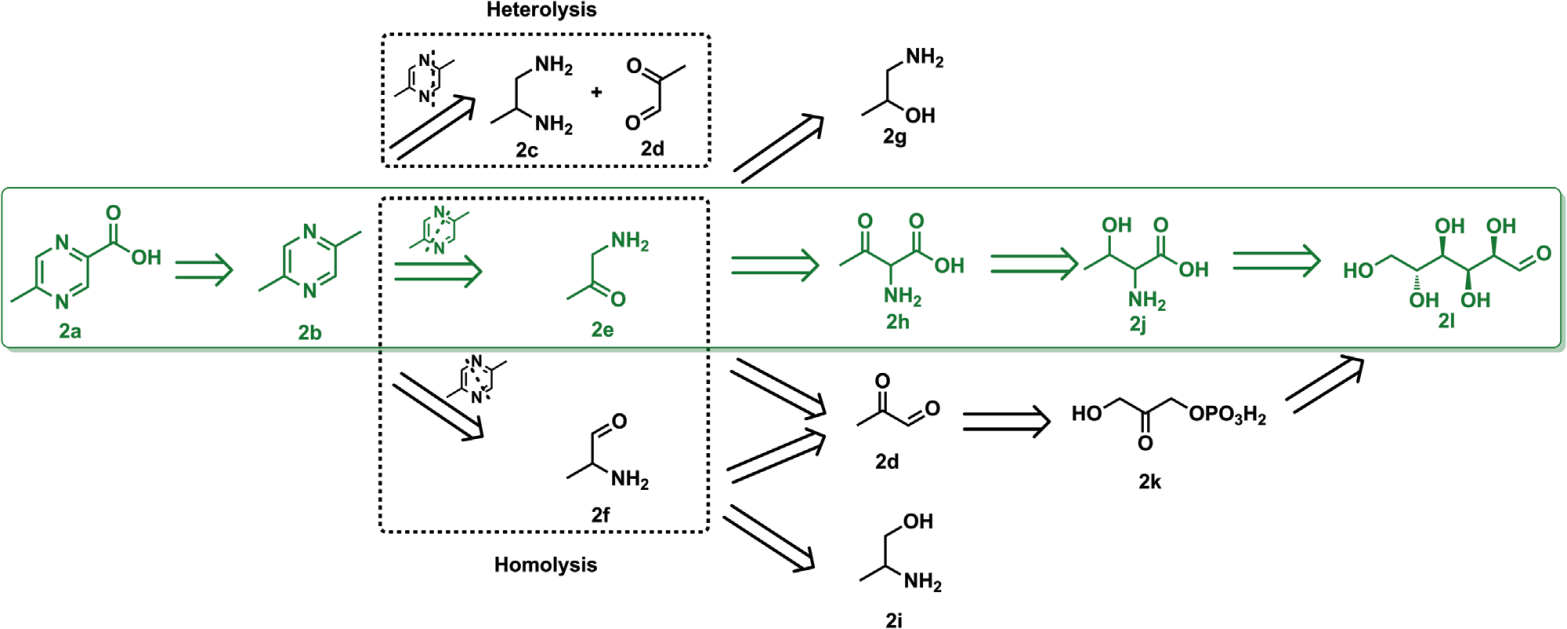
Bioretrosynthetic planning scheme of MPCA leading to central metabolism.

Towards developing an optimal biosynthetic pathway for MPCA (**2a**), we first analyzed different potential routes (**Figure** **2**) from a retrosynthetic perspective. Interconversion of functional groups can be applied to produce the MPCA carboxylate group via the serial oxidation of a methyl group; thus, DMP (**2b**) was selected as the key intermediate. Next, the skeleton of the pyrazine ring was deconstructed. Homolytic routes are clearly simpler than heterolytic routes, wherein two distinct precursor‐supplying pathways (1,2‐diaminopropane (**2c**) and methylglyoxal (**2d**)) must be constructed and matched. The most straightforward way to homolytically obtain the precursors is the oxidation of vicinal amino alcohol (**2g** or **2i**)^[^ ^19^ ^]^ or the transamination of methylglyoxal (**2d**).^[^ ^20^ ^]^ However, it is challenging to construct pathways to produce the corresponding amino alcohols from sugar. Methylglyoxal can be derived via glycolytic pathways, but the corresponding precursor, dihydroxyacetone phosphate (**2k**), is a highly reactive compound that inhibits cell growth.^[^ ^21^ ^]^ Although these direct biotransformations are not ideal, one of the threonine degradation pathways is an effective retrosynthetic scheme. Unlike other l‐amino acid dehydrogenases, threonine dehydrogenase (TDH) targets the hydroxyl group instead of the amino group and catalyses the oxidation of threonine (**2j**) to form 2‐amino‐3‐oxobutyrate (**2h**),^[^ ^22^ ^]^ which undergoes spontaneous decarboxylation to form the DMP precursor 1‐aminoacetone (**2e**). As threonine is a primary metabolite, we engineered biosynthetic and catabolic pathways for this amino acid to appropriate the carbon flux from glucose (**2l**) for the production of *N*‐heterocycles.

Based on the retrosynthetic analysis, we designed three synthetic modules to perform the required biological functions: a module that supplies building blocks by converting sugar into the required threonine; a module wherein *N*‐heterocycles are produced by transforming excess threonine into a *N*‐heterocyclic skeleton; and a functionalization module for the final regio‐selective oxyfunctionalization of pyrazine. These modules can potentially interfere with each other, e.g., consecutive oxidation of heterocycles may significantly shift the redox balance to undermine sugar‐utilization, and inappropriate (de) activation of the functional enzymes can result in insufficient or excessive quantities of a pathway intermediate. To reduce the risk of combining modules with antagonistic effects and achieve optimal productivity for the entire pathway, we used a separate strain for each module.

### 2.2 Construction and Optimization of MPCA Biosynthetic Pathway

**Figure 3:**
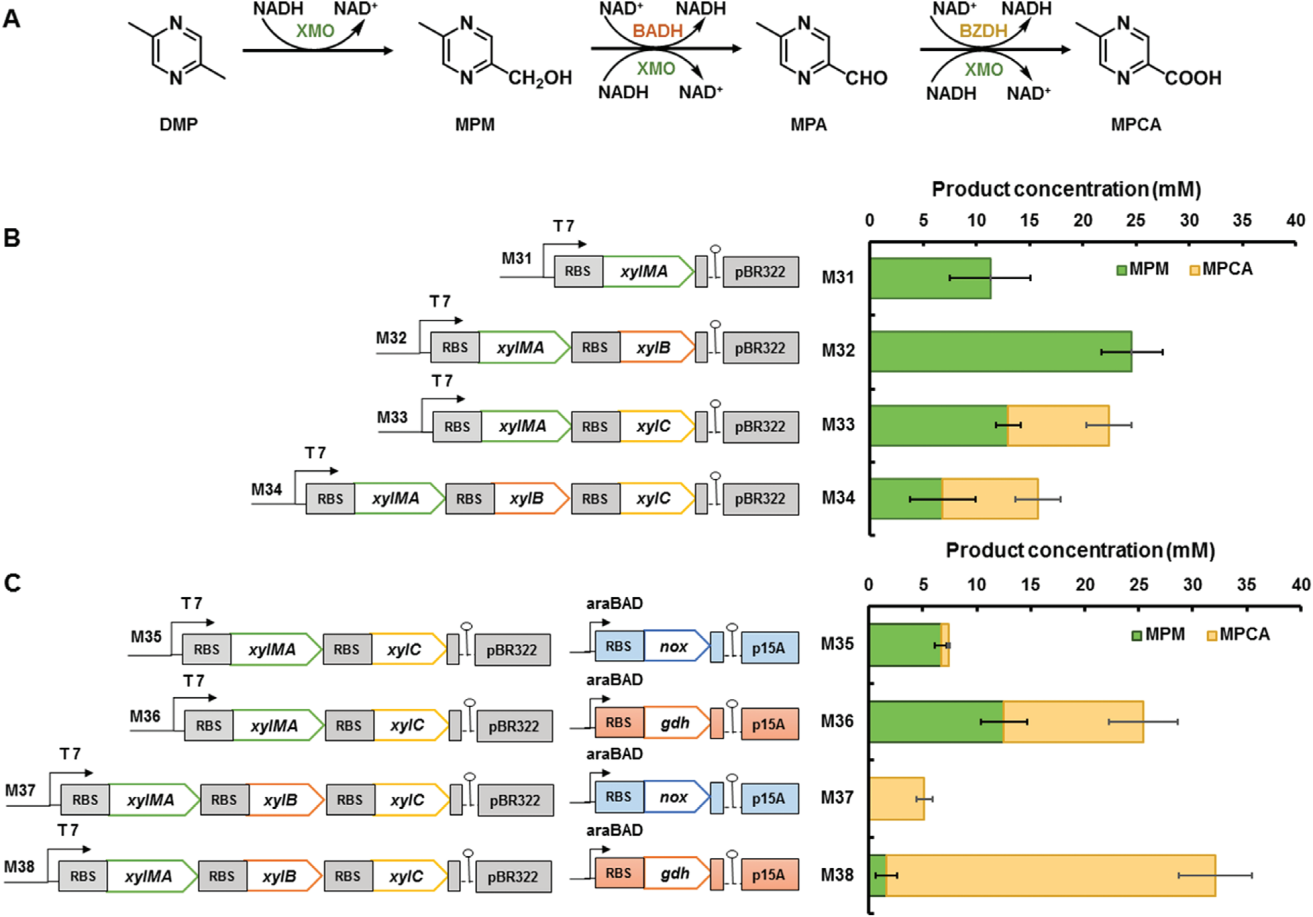
Construction and optimization of MPCA biosynthetic pathway. A) Proposed three‐step MPCA production process utilizing xylene degradation enzymes. B) Schematic of strains harbouring constructed plasmids for MPCA biosynthesis pathway genes expression and product profiles of the strains. C) Schematic of strains containing plasmids for cofactor self‐sufficient systems and product profiles of the strains. Reaction mixture contained 30 × 10^−3^ m DMP in 100 × 10^−3^ m potassium phosphate buffer (pH 7.4). For GDH recycling reactions, 1% w/v glucose was added. The reactions were performed at 30 °C on 200 rpm shaker for 24 h. All plots show mean ± SD for *n* = 3 replicates.

The artificial pathway for MPCA was constructed in reverse. Initially, we used an *Escherichia coli* strain for the *N*‐heterocycle functionalization module. *Pseudomonas putida* is a well‐known degrader of aromatic compounds^[^ ^23^ ^]^ and has been reported to convert DMP to MPCA, although the respective gene cluster has not been elucidated.^[^ ^15^ ^]^ This reaction may occur via xylene degradation, because the degradation product *p*‐toluic acid is a MPCA analogue (**Figure** **3**A). In this pathway, *xylMA*‐encoded xylene monooxygenase (XMO) converts *p*‐xylene to *p*‐tolualcohol, *p*‐tolualdehyde and small amounts of *p*‐toluic acid by sequential oxidation. Additionally, *xylB*‐encoded benzyl alcohol dehydrogenase (BADH) and *xylC*‐encoded benzaldehyde dehydrogenase (BZDH) can oxidize *p*‐tolualcohol and *p*‐tolualdehyde to *p*‐toluic acid.^[^ ^24^ ^]^

Intriguingly, XMO uses NADH as a cofactor, whereas BADH and BZDH oxidize the respective substrates by consuming NAD^+^,^[^ ^24^ ^]^ which could provide redox neutrality for this module. Therefore, four plasmids harboring different combinations of xylene‐degradation genes under a T7 promoter were constructed and transformed into the *E. coli* strain BL21 (DE3) (Figure 3B). The strain functions were examined by assaying the whole‐cell conversion of DMP. Although strain M31 expressing XMO converted DMP as expected, 5‐methyl‐2‐pyrazinemethanol (MPM) was the sole major product. Supplementing strain M32 with BADH significantly increased the MPM yield, although the formation of 5‐methyl‐2‐pyrazinecarbaldehyde (MPA) and MPCA remained negligible (Figure 3B). These results showed the low activity of the endogenous aldehyde dehydrogenases in *E. coli* and that a more efficient enzyme was required to rapidly convert the intermediate MPA to MPCA. Indeed, the deliberate coexpression of BZDH in strains M33 (with XMO) or M34 (with XMO and BADH) produced MPCA (Figure 3B). However, both strains produced significant quantities of the MPM intermediate and lower‐than‐expected MPCA yields.

Considering that the low MPCA yield may have resulted from the absence of an unmatched cofactor for the oxidative enzymes, we introduced additional cofactor recycling enzymes into the system. In the absence of clear evidence of which cofactor to compensate, two opposing types of coordinating enzymes were tested. Glucose dehydrogenase from *B. megaterium* (GDH)^[^ ^25^ ^]^ and NADH oxidase (NOX) from *Streptococcus pyogenes* were coexpressed in the M33 and M34 strains separately (Figure 3C). Unlike the system without supplementary cofactor recycling, XMO, BADH, and BZDH functioned well together, and both NOX and GDH facilitated the reaction towards MPCA as the final product with only trace amounts of MPM. Overall, glucose‐driven cofactor regeneration promoted the reactions. Using strain M38 to coexpress XMO, BADH, BZDH, and GDH produced MPCA from DMP in almost the quantitatively expected amount (Figure 3C). This strain is referred to as the *N*‐heterocycle functionalization strain.

### 2.3 Construction and Optimization of DMP Biosynthetic Pathway

**Figure 4:**
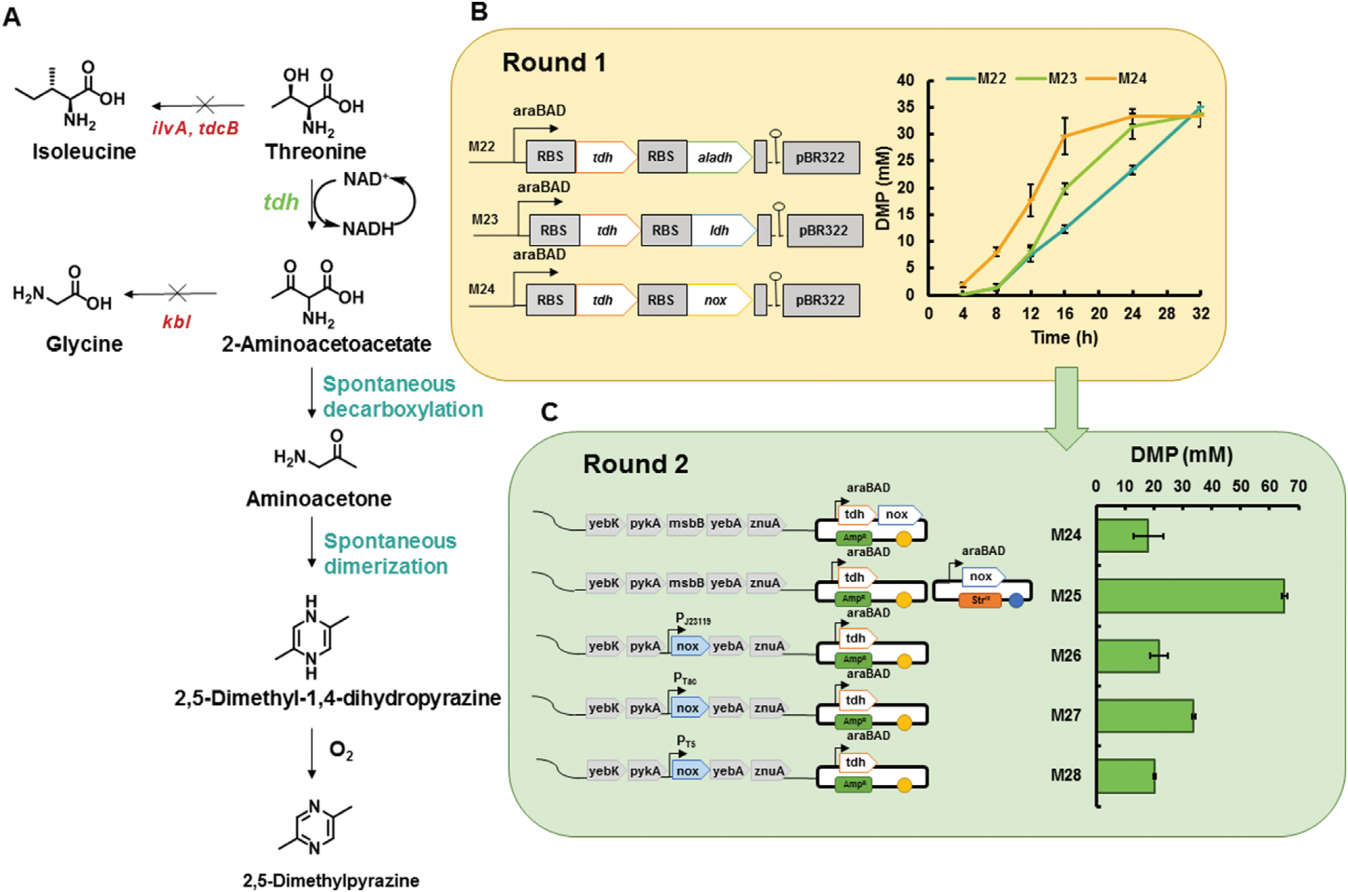
Construction and optimization of DMP biosynthetic pathway. A) Designed synthetic module for DMP formation. B) Round 1 shows whole‐cell biocatalysis for DMP production by strains with different cofactor recycling systems. Reaction mixture contained 200 × 10^−3^ m l‐threonine and 1 × 10^−3^ m NAD^+^ in 0.5 m Tris‐HCl buffer (pH 8.0). 200 × 10^−3^ m pyruvate was added for LDH recycling reactions. 200 × 10^−3^ m pyruvate and 300 × 10^−3^ m ammonia were added for AlaDH recycling reactions. C) Round 2 shows the DMP production of strains harboring different combinations of *Ectdh* and *nox*. Reaction mixture contained 500 × 10^−3^ m l‐threonine and 2 × 10^−3^ m NAD^+^ in 0.5 m Tris‐HCl buffer (pH 8.0). The bioconversion reactions were performed at 30 °C on 200 rpm shaker for 24 h. All plots show mean ± SD for *n* = 3 replicates.

After the construction of the synthetic module for the functionalization of DMP, the next step was to confer the supplying ability of the key building block 1‐aminoacetone. We used a common laboratory strain *E. coli* BW25113 to construct the base strain. Three genes were deleted from the chromosome, namely, *ilvA*, *tdcB* (which both encode threonine dehydratase), and *kbl* (which encodes glycine C‐acetyltransferase) to knock out undesirable pathways that compete with the dehydrogenation of threonine and degrade 1‐aminoacetone, thereby yielding strain M21 (**Figure** **4**A).

Next, we examined the TDHs from *E. coli, B. subtilis*, *Pyrococcus horikoshii*, and *Thermococcus kodakarensis* for use in threonine oxidation. The TDH from *E. coli* exhibited the highest activity (Table S5, Supporting Information) and was selected for further use. As TDH is a NAD^+^‐dependent enzyme, a series of plasmids harbouring genes of TDH and the corresponding cofactor‐regenerating enzyme, including alanine dehydrogenase (AlaDH) from *Vibrio proteolyticus* and lactic dehydrogenase (LDH) from *B. subtilis* or NOX from *S. pyogenes*, were constructed and transformed into strain M21 to yield strains M22, M23, and M24, respectively. We tested the synthetic performance of these strains by measuring DMP production directly, because the 2‐amino‐3‐oxobutyrate and 1‐aminoacetone concentrations were difficult to monitor. All three systems could afford the expected level of DMP (30 × 10^−3^ m) from 200 × 10^−3^ m threonine (Figure 4B), since the chemical condensation of 1‐aminoacetone is known to be unspecific with a typical DMP yield of ≈30%.^[^ ^26^ ^]^ NOX exhibited the best kinetic performance using inexpensive oxygen as the sacrificial substrate and was thus chosen for cofactor recycling.

As increasing the substrate loading decreased the DMP yield (Figure 4C), further optimization efforts are performed to enhance the system resistance to higher substrate concentration. We investigated the enzyme expression profile of strain M24, and the SDS‐PAGE showed that TDH was the dominant enzyme among the expressed soluble proteins (Figure S1, Supporting Information). To de‐bottleneck the threonine oxidation pathway, the expression levels of TDH and NOX must be balanced. Accordingly, we attempted to vary the expression of NOX by transforming another p15A‐derived plasmid (pYB1s) for this enzyme or by introducing the gene for NOX into the chromosome of strain M21 using a constitutive promoter of three different strengths, namely, P_J23119_ (a medium‐strong promoter), P_tac_ (a medium promoter), and P_T5_ (a weak promoter) (Figure 4C). Although the chromosomal expression of NOX was insufficient to increase DMP production, the DMP concentration was significantly increased by coexpressing TDH and NOX using two separate plasmids. This system tolerated threonine concentrations up to 500 × 10^−3^ m to yield 67 ± 1 × 10^−3^ m DMP (Figure 4C). The corresponding strain M25 is referred to as the *N*‐heterocycle‐forming strain.

### 2.4 Construction and Optimization of Threonine Biosynthetic Pathway

**Figure 5:**
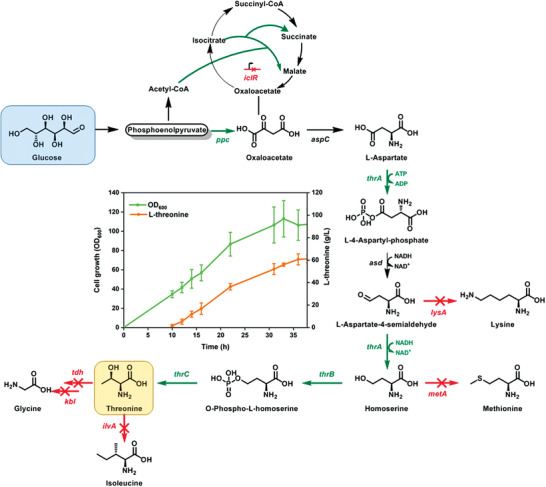
Schematic of engineering strategies for l‐threonine production from glucose and corresponding fed‐batch fermentation profile of strain M11; green line: optical density of the culture; orange line: threonine concentration; thick red arrows marked with cross indicate that the genes are knocked out or the inhibition is removed; thick green arrows indicate the increased flux by directly overexpressing the corresponding genes. All plots show mean ± SD for *n* = 3 replicates.

The last step to complete the MPCA biosynthetic pathway was the construction of a module to provide building blocks to link the downstream modules to glucose. To ensure that this module did not interfere with DMP formation, the native *tdh* (encoding TDH), *ilvA*, and *kbl* genes were deleted from *E. coli* BW25113, similarly as for strain M21. In addition, the following undesirable upstream bypass genes were removed from the chromosome: *lysA* (which encodes diaminopimelate decarboxylase), *metA* (which encodes homoserine succinyltransferase), and the regulatory gene *iclR* (which encodes the regulator of the glyoxylate bypass operon). Subsequently, three key genes *thrA^C1034T^* (encoding aspartokinase I with attenuated feedback inhibition), *thrB* (encoding homoserine kinase), and *thrC* (encoding threonine synthase), which have been reported to enhance the synthetic pathway for threonine,^[^ ^27^ ^]^ were overexpressed in a plasmid under the control of a strong araBAD promoter. To enhance the precursor supply, an additional copy of *ppc* (encoding phosphoenolpyruvate carboxylase) from *Corynebacterium glutamicum* ^[^ ^28^ ^]^ under a P_tac_ promoter was overexpressed. Finally, *rhtC* ^[^ ^29^ ^]^ under a P_J23119_ promoter, which encodes the threonine transporter, were chromosomally overexpressed to enhance the product exportation. Strain M11 was thus obtained. Consequently, we used a fed‐batch culture of this strain with a glucose feed to produce a satisfactory threonine yield of 61.1 ± 3.4 g L^−1^ (0.35 g per gram of glucose) in 36 h (**Figure** **5**). Further optimization was not performed because the downstream module could not tolerate higher threonine concentrations. Strain M11 is referred to as the building‐block‐supplying strain.

### 2.5 Biosynthesis of *N*‐Heterocycles from Sugar Using a Stepwise Culture Strategy

**Figure 6:**
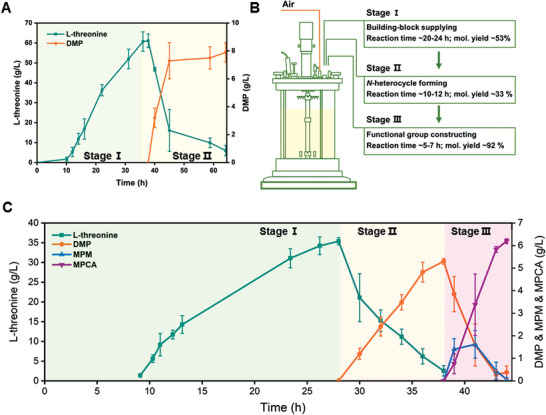
DMP and MPCA biosynthesis from sugar using a stepwise culture strategy. A) Time course of DMP production from glucose. B) Stepwise culture strategy for MPCA production from glucose. C) Time course of MPCA production from glucose. Green line: threonine concentration; orange line: DMP concentration; blue line: MPM concentration; magenta line: MPCA concentration. All plots show mean ± SD for *n* = 3 replicates.

Having constructed all the required strains, we strategically considered the interactions between the designed cells and the most effective compatible reaction scheme for the entire process. We first examined cooperation between the building‐block‐supplying strain (M11) and the *N*‐heterocycle‐forming strain (M25). Strains M11 and M25 were coinoculated in a fermenter under constant glucose feeding. After 47 h of cultivation, the DMP concentration was a mere 14.6 × 10^−3^ m for 200 g L^−1^ of glucose consumption (Figure S2, Supporting Information). Analysis of the fermented coculture composition showed that strain M25 (M25:M11 > 20:1) dominated the consortia. This phenomenon may have resulted from the blocking of multiple essential amino acid synthetic pathways (isoleucine, methionine, and lysine) in strain M11, considerably weakening this strain in the competitive coculture. We prevented microbial competition by developing a spatio‐temporal separation organization for this biotransformation. Here, only strain M11 was inoculated in the initial stage to enable efficient threonine production. When the threonine concentration reached ≈60 g L^−1^, strain M25 was added to the culture to yield 7.9 ± 0.7 g L^−1^ DMP after another 28 h transformation (**Figure** **6**A), which is a remarkably high production level of this odorant compared to that obtained by fermentation with natural strains.^[^ ^30^ ^]^

Next, we studied the compatibility between the *N*‐heterocycle‐forming strain and the functionalization strain (M38). As in the upstream process, more separation was required between strain M25 and strain M38, because strain M38 can also degrade threonine and 1‐aminoacetone, and we failed to knock out the bypass genes from M38 while maintaining the actions of the functionalization module (data not shown). To this end, in the initial trial, we used strain M25 to convert 60 g L^−1^ threonine, and strain M38 was added upon the completion of the DMP formation reaction. Unexpectedly, the conversion of DMP to MPCA did not go beyond ≈55%, although strain M38 could convert much higher concentrations of commercial DMP efficiently. We speculated that the M38 functions may have been inhibited by the spontaneous formation of prevalent by‐products, such as trimethyl‐pyrazine, 2,5‐dimethyl‐3‐ethylpyrazine, and polymerized *N*‐heterocycles. The inhibited yield was moderately improved by decreasing the reactant concentration in the biotransformation (Figure S3, Supporting Information). The target concentration of the final MPCA product was set at 40 × 10^−3^ m to achieve a reasonably high DMP conversion.

Finally, we carried out the complete biotransformation of glucose to MPCA in a bioreactor, and spatial and temporal tuning was realized by adding the necessary cells at the desired stages of the process (Figure 6B). In this setup, strain M11 was initially inoculated in a 1‐L MI medium, and fermentation was performed under glucose‐limited conditions. When threonine production reached ≈35 g L^−1^ at 28 h, the glucose feed was terminated, and strain M25 was added to the bioreactor for threonine conversion. After another 10 h, threonine was almost exhausted, and strain M38 was implemented. The glucose feed was reinitiated, and the glucose concentration was maintained below 1% (*w/v*). The entire one‐pot tandem biotransformation took 44 h, providing a total of 6.2 ± 0.1 g L^−1^ of MPCA with an overall glucose yield of 0.06 g per g glucose (Figure 6C). To the best of our knowledge, this study is the first report of the microbial production of this important pharmaceutical intermediate from a simple sugar and demonstrates the utility and potential of modulated microbial cooperation for the synthesis of xenobiotic high‐value compounds.

## 3 Discussion and Conclusion

Rapid developments in synthetic biology have substantially improved our ability to build microbes with defined functionality and biosynthesize a plethora of compounds of medical and industrial importance.^[^ ^31^ ^]^ Although most of these successes were realized by synergizing various subfunctions in a single microbial strain, challenges arise when specialized environments are required for the optimal functioning for synthetic modules. In recent years, extensive efforts have been expended on the design and construction of microbial cocultures with the desired modularity to perform complex biosynthetic tasks. In addition to the aforementioned classic vitamin C process, major successes to date include the combination of *E. coli* and *Saccharomyces cerevisiae* to produce taxanes,^[^ ^32^ ^]^ the stepwise cultivation of an *E. coli* system for the total synthesis of opiates,^[^ ^33^ ^]^ and using a coculture of *Pichia pastoris* to produce lovastatin.^[^ ^34^ ^]^ Going beyond these lauded studies on reconstructing natural chemicals, artificial microbial consortia are being harnessed to synthesize non‐natural molecules, although the significance of this revolutionary strategy for basic research is yet to be recognized.

Realizing this vision requires the capacity to identify and recruit necessary enzymes from the vast gene bank to act on artificial substrates rather than rebuilding an existing synthetic cluster from plant or fungal to production hosts. Motivated by a recent commentary by Turner,^[^ ^18^ ^]^ researchers are applying the rules of biocatalytic retrosynthesis to generate new routes to synthesize complex organic molecules. In this study, we used a rational process consisting of strategic disconnections of targeted compounds, functional group interconversions and analyses of available enzymes to create a total retrobiosynthetic design for the important pharmaceutical intermediate MPCA. A total of eleven genes to encode synthetic enzymes, transporters, and cofactor‐regenerating enzymes were recruited for overexpression in chromosomes or plasmids. Several key features were required for the efficient implementation of this long biocatalytic cascade: enzymes were grouped into different modules to satisfy the fluctuating redox requirement of the entire transformation and minimize potential regulation, the modules were constructed in separate cells to reduce the burden of genetic modification, and the engineered cells were cultivated sequentially to eliminate growth competition and bypass undesirable reactions in which upstream cells act on downstream compounds. Using these robust and inexpensive cells as catalysts, high‐value‐added *N*‐heterocycles can be readily prepared under mild conditions from cheap glucose substrates in a single aqueous solution without tedious isolation of intermediates. The practical utility of the designed microbial system was demonstrated by scale‐up synthesis of DMP and MPCA following industrially relevant protocols. Furthermore, as the abiotical dimerization of 1‐aminoacetone was identified as the main bottleneck of the entire process, the efficiency of the system could be significantly improved by using a specific bio‐ or chemocatalytic reaction. Hence, the computational rational design of artificial enzymes for amino‐ketone condensation reactions is currently underway in our laboratory.

In summary, we used a retrosynthetic principle to develop a facile microbial system for the synthesis of industrially important *N*‐heterocyclic compounds. The modular nature of our microbial system enabled the plug‐and‐play application of the biocatalysts, resulting in high (>5 g L^−1^) production of the pharmaceutical intermediate MPCA and the odorant DMP. This study highlights the potential of the environmentally benign production of artificial compounds from renewable biomass via biocatalysis and broadens the perspective of designing complex microbial systems for synthetic applications.

## Conflict of Interest

The authors declare no conflict of interest.

## Supporting information

Supporting Information.
