# Supplementary material for: Bioretrosynthesis of Functionalized N‐Heterocycles from Glucose via One‐Pot Tandem Collaborations of Designed Microbes
Source: Adv Sci (Weinh). 2020 Jul 21;7(17):2001188. doi: 10.1002/advs.202001188 (PMC7507072; doi:10.1002/advs.202001188)
Supplement: Supplementary file 1 — Supporting Information. [file ADVS-7-2001188-s001.pdf]

Supporting information

Bioretrosynthesis of functionalized *N*-heterocycles from glucose via one-pot tandem collaborations of designed microbes

Jing Feng[a,b#], Ruifeng Li[a,b#], Shasha Zhang[a,b#], Yifan Bu[a,b], Yanchun Chen[a,b], Yinglu Cui[a], Baixue Lin[a], Yihua Chen[a], Yong Tao[a], Bian Wu[a*].

a. CAS Key Laboratory of Microbial Physiological and Metabolic Engineering, State Key Laboratory of Microbial Resources, Institute of Microbiology, Chinese Academy of Sciences, Beijing 100101, PR China.

b. University of Chinese Academy of Sciences, Beijing, China.

# Equal contribution

* Correspondence to: wub@im.ac.cn

1. General information

Commercial reagents were used as received: L-threonine (shanghai yuanye Bio-Technology), 2,5-dimethylpyrazine (DMP) (Accela ChemBio), 5-methyl-2-pyrazinecarboxylic acid (MPCA) (Accela ChemBio), 5-methylpyrazine-2-carbaldehyde (MPA) (Ark Pharm), (5-methylpyrazin-2-yl) methanol (MPM) (Bide pharm), 2,4-dinitrofluorobenzene (DNFB) (Sigma-Aldrich), Isopropyl-β-D-thiogalactoside (IPTG) (Sigma-Aldrich), L-arabinose (Mackin).

Restriction endonucleases, T4 DNA ligase and Gibson kits were purchased from New England Biolabs (USA). Primer STAR HS DNA polymerase was purchased from Takara Bio Inc. (Beijing,China). Oligo-nucleotides were synthesized by Tsingke (Beijing, China) Co. Ltd.

2. Strains, plasmids and cultures

The details of the strains and plasmids used in this study are given in Supplementary Table 1. Primers used for gene cloning are given in Supplementary Table 2 and primers used for genomic recombination are given in Supplementary Table 3.

Supplementary Table 1. Strains and plasmids

**Strain**	**Description**	**Source**
E. coli BW25113	$lacI^{q}rrnB_{T14}\Delta lacZ_{WJ16}hsdR514\Delta araBAD_{AH33}\Delta rhaBAD_{LD78}$	Invitrogen
E. coli BL21 (DE3)	*F−ompT gal dcm rne131 lon hsdSB (rB_mB_) λ(DE3)*	Invitrogen
M11	*E. coli* BW25113, *ΔompT*::P_{tac}-*Cgppc, ΔiclR, Δtdh, ΔlysA, ΔmetA, ΔilvA*::P_{J23119}-*rhtC, Δkbl*, containing pRBthrA*BC	This study
M21	*E. coli* BW25113 *ΔtdcB,Δkbl, ΔilvA*	This study

M22	Strain M21 containing pBAD-*Ectdh-aladh*	This study
M23	Strain M21 containing pBAD- *Ectdh-ldh*	This study
M24	Strain M21 containing pBAD- *Ectdh-nox*	This study
M25	Strain M21 containing pBAD-*Ectdh*， pYB1s-*nox*	This study
M26	Strain M21, Δ*msbB*::P_{J23119}-*nox*, containing pBAD-*Ectdh*	This study
M27	Strain M21, Δ*msbB*::P_{tac}-*nox*, containing pBAD-*Ectdh*	This study
M28	Strain M21, Δ*msbB*::P_{T5}-*nox*, containing pBAD-*Ectdh*	This study
M31	*E. coli* BL21(DE3) containing pETMA	This study
M32	*E. coli* BL21(DE3) containing pETMAB	This study
M33	*E. coli* BL21(DE3) containing pETMAC	This study
M34	*E. coli* BL21(DE3) containing pETMABC	This study
M35	Strain M33 containing pYB1s-*nox*	This study

M36	Strain M33 containing pYB1s-*gdh*	This study
M37	Strain M34 containing pYB1s-*nox*	This study
M38	Strain M34 containing pYB1s-*gdh*	This study
**Plasmid**	**description**	**source**
pBAD	araBAD promoter, pBR322 ori, Amp^R	Our lab
pET21a	T7 promoter, pBR322 ori, Amp^R	Our lab
pET28b	T7 promoter, pBR322 ori, Kan^R	Our lab
pRB1k	araBAD promoter, RSF1030 ori, Kan^R	Our lab
pYB1s	araBAD promoter, p15A ori, Str^R	Our lab
pYB1s-*nox*	pYB1s carrying *nox* from *S. pyogenes*	Our lab
pYB1s-*gdh*	pYB1s carrying *gdh* from *B. megaterium*	Our lab
pS95s-nox	J23119 promoter, pSC101 ori, Str^R, carrying *nox* from *S. pyogenes*	Our lab
pSC2s-nox	tac promoter, pSC101 ori, Str^R, carrying *nox* from *S. pyogenes*	Our lab
pST3s-nox	T5 promoter, pSC101 ori, Str^R, carrying *nox* from *S. pyogenes*	Our lab
pS95s-rthC	J23119 promoter, pSC101 ori, Str^R, carrying *rthC* from *E. coli*	Our lab

pSC2s-Cgppc	tac promoter, pSC101 ori, Str^R, carrying *ppc* from *C. glutamicum*	Our lab
pRBthrA*BC	pRB1k carrying *thrA*^{C1034T}*BC* from *E. coli*, with a mutant C1034T in *thrA*	This study
pBAD-*Ectdh*	pBAD carrying *tdh* from *E. coli*	This study
pBAD-*Bstdh*	pBAD carrying *tdh* from *B. subtilis*	This study
pET28b-*Tktdh*	pET28b carrying *tdh* from *T. kodakaraensis*	This study
pET28b-*Phtdh*	pET28b carrying *tdh* from *P. horikoshii*	This study
pBAD-*nox*	pBAD carrying *nox* from *S. pyogenes*	This study
pBAD-*aladh*	pBAD carrying *aladh* from *V. proteolyticus*	This study
pBAD-*ldh*	pBAD carrying *ldh* from *B. subtilis*	This study
pBAD-*Ectdh-nox*	pBAD-*Ectdh* carrying *nox* from *S. pyogenes*	This study
pBAD-*Ectdh-aladh*	pBAD-*Ectdh* carrying *aladh* from *V. proteolyticus*	This study
pBAD-*Ectdh-ldh*	pBAD-*Ectdh* carrying *ldh* from *B. subtilis*	This study

pETMA	pET21a carrying *xylM*, *xylA* from *P. putida*	This study
pETMAB	pET21a carrying *xylM*, *xylA*, *xylB* from *P. putida*	This study
pETMAC	pET21a carrying *xylM*, *xylA*, *xylC* from *P. putida*	This study
pETMABC	pET21a carrying *xylM*, *xylA*, *xylB*, *xylC* from *P. putida*	This study

Supplementary Table 2. Primers for cloning

Primers	Sequence (5'-3') [a]
thrABC-NcoI-F	GGCTAACAGGAGGAATTAA**CCATGC**GAGTGTTGAAGTTCGG
thrABC-EcoRI-R	GCTGCAGACCGAGCTCACC**GAATTC**TTACTGATGATTCATCATCA
thrA^{C1034T}-F	CACCACGAAAATACGGGCGCGTGACATCG
thrA^{C1034T}-R	CGTATTTTCGTGGTGCTGATTACGCAATC
pBAD-F	AGCTTGGGCCCGAACAAAAACTC
pBAD-R	ATGGCTGCCGCGCGGCACCAGG
EcTDH-F	CCTGGTGCCGCGCGGCAGCCATATGAAAGCGTTATCCAAACTGAAAGCGG
EcTDH-R	GAGTTTTTGTTCGGGCCCAAGCTTTAATCCCAGCTCAGGATAACTTTCCCGG
BsTDH-F	CTGGTGCCGCGCGGCAGCCATATGCAGAGTGGAAAGATGAAAGCTCT

	AATG
BsTDH-R	GAGTTTTTGTTCGGGCCCAAGCTTTATGGAATTAAAATTACTTTTCCG CAC
LDH-F	CCTGGTGCCGCGCGGCAGCCATATGATGAACAAACATGTAAATAAAG
LDH-HindIII-F	GGGATTA**AAGCTT**CTAACAGGAGGAATTAACCATGATGAACAAACAT GTAAATAAAG
LDH-SalI-R	GATGATG**GTCGAC**TTAGTTGACTTTTTGTTCTGCAAAATGAG
AlaDH-HindIII-F	GGGATTA**AAGCTT**CTAACAGGAGGAATTAACCatgATCATTGGCGTAC CTAAGGAAATC
AlaDH-SalI-R	GATGATGATG**GTCGAC**TTAGTTGAACATGGCGATCGCGG
NOX-HindIII-F	GGGATTA**AAGCTT**CTAACAGGAGGAATTAACCatgGAGTCTAAAATCG TTGTCGTAG
NOX-SalI-R	GATGATGATG**GTCGAC**TTAGTCTTTAGCACCCAGAGCCGCC
NOX-F	CTAACAGGAGGAATTAACCATGGAGTCTAAAATCGTTGTCGT
pET21a-F	GGATCCGAATTCGAGCTCCGTC
xylma-R	GACGGAGCTCGAATTCGGATCCTAGCAAGGAGGTCTATTATAAAAAC GGTCC
xylmab-R	GACGGAGCTCGAATTCGGATCCTCAACCAATCCGGAGTACCGGCTTA AG
xylmac-F	GTTTTTATAATAGACCTCCTTGCTAGATCGATTGCCACTATCGGCGG
xylmac-R	CCGCCGATAGTGGCAATCGATCTAGCAAGGAGGTCTATTATAAAAAC

[a]Restriction sites are shown in boldface.

Supplementary Table 3. Primers for genomic recombination

Primers	Sequence (5'-3')
lysA-up-F	CTGCGTTGGTCGTCCATGCCAAAATG
lysA-up-R	GAAGAATTACTGGCGCTGGAATTGC
lysA-down-F	CAATTCCAGCGCCAGTAATTCTTCGTATCGGTGCTGAACAGTGAATGTG
lysA-down-R	GAGTCCTAAATCATGACGCTGGGCC
pT-lysA-F	CAATTTCCAGTTTCACAGGGGTTTTAGAGCTAGAAATAGCAAG
pT-lysA-R	CCCTGTGAAACTGGAAATTGACTAGTATTATACCTAGGAC
metA-up-F	GAACCCAACCGCCTGCTCATTTTGC
metA-up-R	CTCGTCCGGCACACGAATCGGCAT
metA-down-F	TGCCGATTCGTGTGCCGGACGAGGATCTACGGCACATGAATCCAAC
metA-down-R	CTCTACGCGGCGGTCTTCTAAGTCC
pT-metA-F	ACTCGCACCGAAAAACTCTCGTTTTAGAGCTAGAAATAG
pT-metA-R	GAGAGTTTTTCGGTGCGAGTACTAGTATTATACCTAGGACTG
ilvA-up-F	CACCGGCCCGGCGAAAGTGTACGAAAG
ilvA-up-R	CACCGGACAGGGGTTGCGAGTCAGCC
ilvA-down-F	GACTCGCAACCCCTGTCCGGTGATAACCCGGCGTTCAGGTTCTTTTTGG
ilvA-down-R	GGTAAACCGGAAACCTTGCCCGGC
ilvA-ppc-R	CAGCAGATAGGGACGACGTGGTGTTAGCTGTGCACCGGACAGGGGTTGCGAGTC
ilvA-ppc-F	TCGATATCGCAGGTGAGTACTATAACCCGGCGTTCAGGTTCTTTTTGG
pT-ilvA-F	CGATAGCGATGCGATCTGTGGTTTTAGAGCTAGAAATAG

pT-ilvA-R	CACAGATCGCATCGCTATCGACTAGTATTATACCTAGGACTGAG
kbl-up-F	GTTGCCACTTCAATCCCACGATTACGCGCC
kbl-up-R	TTATCTATTGGTAAACAACTGGGCGTTATC
kbl-down-F	AGTTGTTTACCAATAGATAAAATTCTCCACGCATTGCGATTCTCC
kbl-down-R	ATGGAAAGAATTCTATAAATTAGTTCTGG
pT-kbl-F	CAACAACGATATGCAGGAGCGTTTTAGAGCTAGAAATAGCAAG
pT-kbl-R	GCTCCTGCATATCGTTGTTGACTAGTATTATACCTAGGACTG
tdh-up-F	CACTTCAATCCCACGATTACGCGCC
tdh-up-R	GGAAAGTTATTCTGAGCTGGGATTAAAC
tdh-down-F	CCCAGCTCAGAATAACTTTCCCCGCTTTCAGTTTGGATAACGCTTTC
tdh-down-R	GGCGCGCAAAGAAGTGGTTGAGTGG
pT-tdh-F	CAGTCGATAGACATATCAGAGTTTTAGAGCTAGAAATAG
pT-tdh-R	TCTGATATGTCTATCGACTGACTAGTATTATACCTAGGACTGAG
iclR-up-F	CTTGTAAGACGGACGTGGAAAATTTAATG
iclR-up-R	CTGGCGTACGGTGGAATGCGCTG
iclR-down-F	CAGCGCATTCCACCGTACGCCAGCGGGAATGGGTGCGACCATACAGTC
iclR-down-R	GTGGAGTTGAAGGTGTTGGTTTCGATGATATC
pT-iclR-F	GGTTAGCAGGCGGTGGGTCGGTTTTAGAGCTAGAAATAG
pT-iclR-R	CGACCCACCGCCTGCTAACCACTAGTATTATACCTAGGACTG
ompT-up-F	CAGTTTTAATATTGAGCGGCAATGG
ompT-up-R	GGACGACGTGGTGTTAGCTGTGAACGCCAACTAAAATTTCCC

	CGAGGTG
ompT-down-F	GATATCGCAGGTGAGTACTAAAGTTCTCCATTCAATCGTTTTAATG
ompT-down-R	GGAAACGGATAAGACGGGCATAAATGAGG
pT-ompT-F	GCAGCCCCGATAGATATCTGGTTTTAGAGCTAGAAATAG
pT-ompT-R	CAGATATCTATCGGGGCTGCACTAGTATTATACCTAGGACTGAG
tdcB-up-F	GTTCCAGTAAGGGATCAGCGACAAAG
tdcB-up-R	CTCTCAAATCACCGGTTTCGTTGAC
tdcB-down-F	CGAAACCGGTGATTTGAGAGGTCATCAATAGCAACCGGCAGATC
tdcB-down-R	GATTTACATGTTGAGCCGCTGTTCGAGTC
pT-tdcB-F	CGTAAGTTAAATTACCCGGGGTTTTAGAGCTAGAAATAGCAAG
pT-tdcB-R	CCCGGGTAATTTAACTTACGACTAGTATTATACCTAGGACTG
msbB-up-F	CCGCGTCGTACTAATCCGGTG
msbB-up-R	CGACGTGGTGTTAGCTGTGCAACTTGCCCCGCACCAAA
pT-msbB-F	ATATCGACGGCCCAACCGTGGTTTTAGAGCTAGAAATAG
pT-msbB-R	CACGGTTGGGCCGTCGATATACTAGTATTATACCTAGGACTG
msbB-down-F	GATATCGCAGGTGAGTACTAAAGCCTCTCGCGAGGAGAG
msbB-down-R	AACACCGTCTGGACGTTCAG
tac/J25119/T5-F	CACAGCTAACACCACGTCG
rrnB-R	AGTACTCACCTGCGATATC

Luria-Bertani (LB) medium (per liter: 10 g tryptone, 5 g yeast extract and 10 g NaCl) was used for all molecular cloning experiments and small-scale cultivations. The cultures used for high-density fermentations are summarized in Supplementary Table 4.

When needed, ampicillin, streptomycin and kanamycin were used at final concentrations of 50 mg/L.

Supplementary Table 4. Culture conditions of high-density fermentation.

	Medium composition (per liter)	Nutrient feeding solution (per liter)
MI medium	Minimal medium①, 20 g of glucose, 0.4 g of Lys, 0.2 g of Met, 0.2 g of Ile, 5 g of yeast extract, 10 g of tryptone.	60 % (*w/v*) glucose, 10 mL Trace elements solution②, 3.0 g of Lys, 1.5 g of Met, 1.5 g of Ile, 2.5 g $MgSO_4 \cdot 7H_2O$
MII medium	Minimal medium①, 20 g of glucose, 0.3 g of Ile, 1 g of yeast extract.	60 % (*w/v*) glucose, 10 mL Trace elements solution②, 1.0 g of Ile, 2.5 g $MgSO_4 \cdot 7H_2O$
MIII medium	Minimal medium①, 10 g of glucose, 5 g of yeast extract, 10 g of tryptone.	60 % (*w/v*) glucose, 10 mL Trace elements solution②, 2.5 g $MgSO_4 \cdot 7H_2O$

Minimal medium① (per liter): 14 g KH_2PO_4, 4 g $(NH_4)_2HPO_4$, 1.4 g citric acid, 0.6 g $MgSO_4 \cdot 7H_2O$, 10 mL trace elements solution①.

Trace elements solution① (per liter): 840 mg EDTA, 250 mg $CoCl_2 \cdot 6H_2O$, 1500 mg/L $MnCl_2 \cdot 4H_2O$, 150 mg $CuCl_2 \cdot 2H_2O$, 300 mg H_3BO_3, 250 mg $Na_2MoO_4 \cdot 2H_2O$, 1300 mg Zn $(CH_3COO)_2 \cdot 2H_2O$, and 10 g ammonium ferric citrate.

Trace elements solution② (per liter): 1300 mg EDTA, 400 mg $CoCl_2 \cdot 6H_2O$, 2350 mg $MnCl_2 \cdot 4H_2O$, 250 mg $CuCl_2 \cdot 2H_2O$, 500 mg H_3BO_3, 400 mg $Na_2MoO_4 \cdot 2H_2O$, 1600 mg Zn $(CH_3COO)_2 \cdot 2H_2O$, and 4 g ammonium ferric citrate.

3. Analytical methods

High-performance liquid chromatography (HPLC) was performed on a LC-2030C HT system (SHIMADZU, Japan) equipped with a C18AQ column (4.6 × 250 mm, 5 μm, SilGreen, Beijing, China). For the mobile phase, buffer A (0.1% formic acid in distilled water) and buffer B (acetonitrile) was used at a flow rate of 1 mL/min. The column temperature was maintained at 40 °C. Determination of DMP, MPM, MPA and MPCA were achieved through an isocratic elution with a mixture of 10% buffer B and 90% buffer A at UV 275 nm. The L-threonine was determined with an elution of 35% buffer B at UV 360 nm after DNFB derivatization.

Derivatization: A mixture of 25 µL amino acid standard solutions or reaction solutions (concentration of amino acid < 50 mM) were mixed with 10 µL 1 M $NaHCO_3$, 40 µL DNFB (36.8 mM in acetone) and incubated at 60 °C for 30 min. The reaction was stopped by adding 20 µL 1 M HCl and the precipitates were subsequently removed by centrifugation.

4. Construction and optimization of the MPCA biosynthetic pathway

a) DNA manipulation

The plasmid pETMABC containing genes of *xylM*, *xylA*, *xylB* and *xylC* was synthesized and constructed by General Biosystems (Anhui) Co. Ltd. To construct the plasmid pETMA, the fragment of pET21a-xylMA was amplified from pETMABC using primes pET21a-F and xylma-R. To construct the plasmid pETMAB, the fragment of pET21a-xylMAB was amplified from pETMABC using primers pET21a-F and xylmab-R. To construct the plasmid pETMAC, the fragment of xylC-pET21-xylMA was amplified from pETMABC using primers xylmac-F and xylmac-R. The amplified and purified fragments were self-joined by the Gibson assembly method[1] and transformed into *E. coli* TOP10. Positive clones were selected on LB agar plate supplemented with ampicillin (50 mg/L) and confirmed by sequencing (Tsingke, Beijing, China).

b) Whole-cell biocatalysis for *N*-heterocycles functionalization

For MPCA bioconversion, whole-cell assays were carried out using *E. coli* BL21 (DE3) transformed with different plasmids. Cells were cultivated in 50 mL LB medium containing appropriate antibiotics at 37 °C with shaking at 200 rpm. When the optical density (OD) at 600 nm of the medium reached 1.0, 1 mM IPTG was added to the cultures to induce the expression of recombinant proteins (0.2 % (*w/v*) L-arabinose was added when the strain containing a plasmid pRB1s) and the cells were cultivated at 30 °C for 2 h. The induced cells were collected by centrifugation and resuspended in 1 mL reaction mixture (4.8 g/L dry cell weight (DCW)) containing 30 mM DMP and 100 mM potassium phosphate buffer (pH 7.4). For GDH recycling reactions, 1% (*w/v*) glucose was added. The bioconversion reactions were performed at 30 °C with shaking at 200 rpm for 24 h. The concentrations of the *N*-heterocycles were determined by HPLC analysis as described above.

5. Construction and optimization of the DMP biosynthetic pathway

a) DNA manipulation and genome editing

Plasmids pET28b-*Phtdh*, pET28b-*Tktdh* and pBAD-*aladh* were synthesized and constructed by General Biosystems (Anhui) Co. Ltd. To construct the plasmid pBAD-*Ectdh*, gene *Ectdh* was amplified from the genome of *E.coli* BW25113 genome with primers EcTDH-F and EcTDH-R. The pBAD vector was amplified using primers pBAD-F and pBAD-R primers. The amplified *Ectdh* was cloned into pBAD vector at the *Nde* I and *Hind* III sites via Gibson assembly method[1]. Plasmids pBAD-*Bstdh*, pBAD-*ldh*, pBAD-*nox* were constructed analogously. Gene *Bstdh* was amplified from genome of *B. subtilis 168* with primers BsTDH-F and BsTDH-R. Gene *ldh* was amplified from *B. subtilis 168* genome using the primers LDH-F and LDH-SalI-R. Gene *nox* was amplified from pRB1s-*nox* using primers NOX-F and NOX- SalI-R.

To construct plasmid pBAD-*Ectdh*-*aladh*, the RBS-*aladh* cassette amplified from pBAD-*aladh* using the primers AlaDH-HindIII-F and AlaDH-SalI-R, was cloned into pBAD-*Ectdh* at the *Hin*d III and *Sal* I sites. Plasmids pBAD-*Ectdh*-*ldh* and pBAD-*Ectdh*-*nox* were constructed analogously. The RBS-*ldh* cassette was amplified from pBAD-*ldh* using the primers LDH-HindIII-F and LDH-SalI-R, and the RBS-*nox* cassette was amplified from pBAD-*nox* using the primers NOX-HindIII-F and NOX-SalI-R.

For genome editing, including gene deletion and gene integration. Strain M21 was obtained via deletion of locus *ilvA*, *tdcB*, *kbl* in the genome of *E. coli* BW25113 using CRISPR-Cas9 system[2]. The fragments of J25119-nox-rrnB, tac-nox-rrnB and T5-nox-rrnB were amplified from pS95s-nox, pSC2s-nox and pST3s-nox, respectively. Then the fragments were assembled with 500 nt of upstream/downstream region of gene msbB by overlap-extension PCR, resulting in the donor DNA fragments for gene integration. To obtain mutant strains, gene integration was carried out using the CRISPR-Cas9 system as well. The mutant strains were verified by PCR and further confirmed by sequencing (Tsingke, Beijing, China). Then plasmid pBAD-*Ectdh* was transformed into the mutant strains to obtain strain M26, M27, and M28 respectively.

b) Overexpression and purification of TDHs

The pBAD-*Ectdh* and pBAD-*Bstdh* were transformed into *E. coli* BW25113 for L-arabinose-induced expression. The pET28b-*Phtdh* and pET28b-*Tktdh* were transformed into *E. coli* BL21 (DE3) for IPTG-induced expression. The transformed cells were cultivated in 5 mL LB medium containing antibiotics at 37 °C for 12 h with shaking. Then the cells were inoculated into 500 mL LB medium. When OD_{600} reached 1.0, the production of the recombinant protein was induced by adding 1 mM IPTG or 0.2 % (*w/v*) L-arabinose. After 16 h cultivation at 30 °C, cultures were collected by

centrifugation at 6,000 × g for 15min. The collected cells were lysed by sonication in a buffer containing 50 mM Tris-HCl (pH 7.5). After centrifugation (10,000 × g, 1 h) and filtration (0.22-μm filter, Millex), the supernatant was loaded onto a pre-equilibrated 5-mL HisTrap HP column (GE Healthcare, Milwaukee, WI, USA). After washed with binding buffer containing 20 mM imidazole, the protein-bound column was eluted with elution buffer containing 300 mM imidazole, then protein eluate was desalted by a HiPrep 26/10 Desalting column (GE Healthcare, USA). The enzyme solution was stored in 50 mM Tris-HCl (pH 7.5) at -80 °C for further using. Protein concentrations were determined with the Protein Quantification Kit (BCA Assary) (Abbkine, Beijing, China).

c) Enzyme assays of TDHs

TDH activities were determined by measuring the formation of NADH, following the absorbance at 340 nm using a UV-1800PC spectrophotometer (MAPADA, Shanghai, China) at 30 °C. The 1-mL reaction mixture contained 50 mM Tris-HCl buffer (pH 8.0), 10 mM L-threonine and 1 mM NAD^+. In the assay, the reaction was initiated by the addition of 5 μg enzyme. The extinction coefficient for NADH of 6.22 $mM^{-1}cm^{-1}$ at 340 nm was used. The specific activities of different TDHs were summarized in Supplementary Table 5.

Supplementary Table 5. Expression system and activities of TDHs from different organisms.

Enzyme	Expression system	Activity (mU/mg)
PhTDH	pET28b/ BL21 (DE3)	32 ± 4
TkTDH	pET28b/ BL21 (DE3)	58 ± 6
BsTDH	pBAD/ BW25113	387 ± 52
EcTDH	pBAD/ BW25113	6446 ± 1335

One unit (U) of enzymatic activity corresponds to the formation of 1 μmol of NADH per min.

d) Whole-cell bioconversion for DMP production

Strains M21 harboring pBAD-*Ectdh-aladh*, pBAD-*Ectdh-ldh* and pBAD-*Ectdh-nox* were incubated in 50 mL LB medium supplemented with 50 mg/L ampicillin at 37 °C. Cells were induced with 0.2 % (*w/v*) L-arabinose when OD_{600} reached 1.0. After 12 h of induction, cells were collected and suspended in 1 mL reaction mixture (12 g/L DCW) containing different concentrations of L-threonine and NAD^+ in 0.5 M Tris-HCl buffer (pH 8.0). 200 mM pyruvate was added for LDH recycling reactions. 200 mM pyruvate and 300 mM ammonium chloride were added for AlaDH recycling reactions. The bioconversion reactions were performed at 30 °C with shaking at 200 rpm. The concentrations of L-threonine and DMP were separately determined by HPLC analysis as described above.

e) SDS-PAGE analysis of protein expression profile of strain M24

The induced M24 cells were lysed by sonication in a buffer containing 50 mM Tris-HCl (pH 7.5), and disrupted cells were removed by centrifuging at 12,000 × g for 5 min at 4 °C. The supernatant was used for SDS-PAGE analysis.

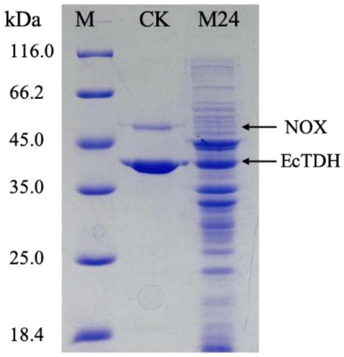

Supplementary Figure 1. SDS-PAGE of *E. coli* strain M24. Line M, molecular-mass protein marker; line CK, purified recombinant enzymes of EcTDH (~37 kD) and NOX (~50 kD); line M24, cell-free extract of strain M24.

6. Construction of L-threonine biosynthetic pathway

DNA manipulation and genome editing

To obtain strain M11, the deletion of the *iclR*, *tdh*, *lysA*, *metA*, *kbl* in *E. coli* BW25113 was performed using the CRISPR-Cas9 system. The cassettes of J23119-rhtC-rrnB and tac-Cgppc-rrnB were amplified from pS95s-rhtC and pSC2s-Cgppc, respectively. Then, cassettes were assembled with about 500 nt of the upstream/downstream region of target genes by overlap-extension PCR, resulting in the donor DNA fragments for gene integration. The integration was carried out using the CRISPR-Cas9 system as well.

The plasmid pRB1k used for gene expression was derived from expression vectors previously developed in our laboratory (unpublished) and have the following features: a promoter (araBAD), multiple cloning sites, rrnB terminator, origin of replication RSF1030, and kanamycin resistance genes. The plasmid pRBthrA*BC was constructed by introducing the *thrA*^{C1034T}*BC* operon of *E. coli* into the *Nco* I and *Eco*R I sites of pRB1k by the Gibson assembly method [1].

7. The fed-batch fermentation

a) The fed-batch fermentation of M11

A single colony of strain M11 from a fresh LB-agar plate was used to prepare a seed culture by inoculation of 50 mL LB broth in a 250 mL flask, followed by cultivation to OD_{600}= 1 at 37 °C. A seed culture was inoculated 5% (*v/v*) into a 2-L fermenter (Bailun Bio, shanghai, China) containing 1 L MI medium. The fermentation inoculation was started with an initial glucose concentration of 20 g/L at 37 °C. MI nutrient feeding solution was manually added to keep the growth of cells when glucose became exhausted indicated by a sudden increase of DO. When the OD_{600} reached approximately 30, 0.2 % (*m/v*) L-arabinose was added to induce the expression of the *thrA*, *thrB*, and *thrC*. During the whole process, the pH was kept at 7.0 by the feeding of 25% (*v/v*) ammonia solution. The DO level was kept at 30% of air saturation by controlling the cascading impeller speed. The aeration rate was kept at 1.0 *v/v·m* . Samples were taken periodically for measurement of cell density and metabolite analysis.

b) The fed-batch fermentation of M25

A single colony of strain M25 from a fresh LB-agar plate was used to prepare a seed culture by inoculation of 50 mL LB broth in a 250 mL flask, followed by cultivation to OD_{600}= 1 at 37 °C. A seed culture was inoculated 5% (*v/v*) into a 2-L fermenter (Bailun Bio, shanghai, China) containing 1 L MII medium. The fermentation inoculation was started with an initial glucose concentration of 20 g/L at 37 °C. MII nutrient feeding solution was manually added to keep the growth of cells when glucose became exhausted indicated by a sudden increase of DO. When the OD_{600} reached approximately 30, temperature was adjusted to 30 °C, 0.2 % (*m/v*) L-arabinose was added to induce and the fermentation continued for another 8 h. During the whole process, the pH was kept at 7.0 by the feeding of 25% (*v/v*) ammonia solution. The DO level was kept at 30% of air saturation by controlling the cascading impeller speed. The

aeration rate was kept at 1.0 *v/v·m*. The induced cells were collected by centrifugation and stored at 4 °C for further use.

c) The fed-batch fermentation of M38

A single colony of strain M38 from a fresh LB-agar plate was used to prepare a seed culture by inoculation of 50 mL LB broth in a 250 mL flask, followed by cultivation to OD_{600}= 1 at 37 °C. A seed culture was inoculated 5% (*v/v*) into a 2-L fermenter (Bailun Bio, shanghai, China) containing 1 L MIII medium. The fermentation inoculation was started with an initial glucose concentration of 10 g/L at 37 °C. MIII nutrient feeding solution was manually added to keep the growth of cells when glucose became exhausted indicated by a sudden increase of DO. When the OD_{600} reached approximately 20, temperature was adjusted to 30 °C, 0.5 mM IPTG and 0.2 % (*m/v*) L-arabinose were added to induce, and the fermentation continued for another 2 h. During the whole process, the pH was kept at 7.0 by the feeding of 25% (*v/v*) ammonia solution. The DO level was kept at 30% of air saturation by controlling the cascading impeller speed. The aeration rate was kept at 1.0 *v/v·m*. The induced cells were collected by centrifugation for further use.

8. Biosynthesis of *N*-heterocycles from sugar using a stepwise culture strategy

a) The co-culture fermentation of strains M11 and M25

Single colonies of M11 and M25 were inoculated in 50 mL LB medium at 37 °C separately. Two seed cultures were inoculated into a 2-L fermenter containing 1 L MI medium at ratio of 2:1 (M11:M25). The fermentation inoculation was started with an initial glucose concentration of 20 g/L at 37 °C. MI nutrient feeding solution was manually added to keep the growth of cells when glucose became exhausted indicated by a sudden increase of DO. When the OD_{600} reached approximately 40, 0.2 % (*m/v*) L-arabinose was added to induce and the concentrations of L-threonine and DMP were monitored with HPLC. During the whole process, the pH was kept at 7.0 by the feeding

of 25% (*v/v*) ammonia solution. The DO level was kept at 30% of air saturation by controlling the cascading impeller speed. The aeration rate was kept at 1.0 *v/v·m*.

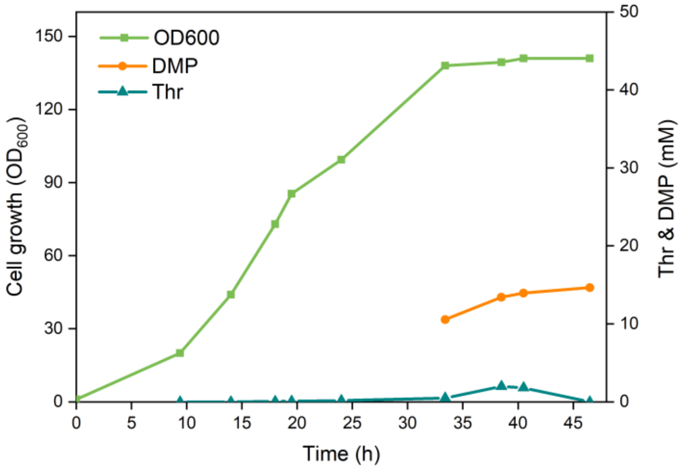

Supplementary Figure 2. Time profile of cell growth, L-threonine accumulation, DMP production during the fed-batch culture of co-culture system of strain M11 and M25.

The real-time cell-to-cell ratio of the two components in the co-culture was analyzed by resistance screening. Strain M11 contained a plasmid with kanamycin resistant gene, whereas strain M25 contained a plasmid with ampicillin resistant gene. To conduct resistance screening, the co-culture sample was diluted 10^5- to 10^6-fold before being spread onto LB agar plates with 50 mg/L kanamycin or 50 mg/L ampicillin. After 24 h cultivation at 37 °C, the phenotypic screening was conducted by counting colonies on each plate.

b) The sequential biocatalysis of strains M11 and M25

Fed-batch fermentation with strain M11 was conducted with the protocols similar to that described above. After 36 h of fermentation, the concentration of L-threonine reached 62.7 g/L. Then the nutrient feeding solution delivering was stopped and the process of

Stage I was complete. The pH value was maintained at 8.0 by feeding 4 M NaOH for the biotransformation. The collected cells of strain M25 (12 g/L DCW) together with 2 mM NAD^+ were added to the culture to initiate the Stage II conversion (L-threonine to DMP). The concentrations of L-threonine and DMP were monitored with HPLC.

c) The sequential biocatalysis of strains M25 and M38

The biotransformations of M38 were conducted with the protocols similar to that described above. The substrates used in the reactions were either commercial DMP (analytical purity) or the reaction mixture derived from M25-catalyzed reactions to generate DMP. The bioconversions were performed at 30 °C with shaking at 200 rpm for 24 h.

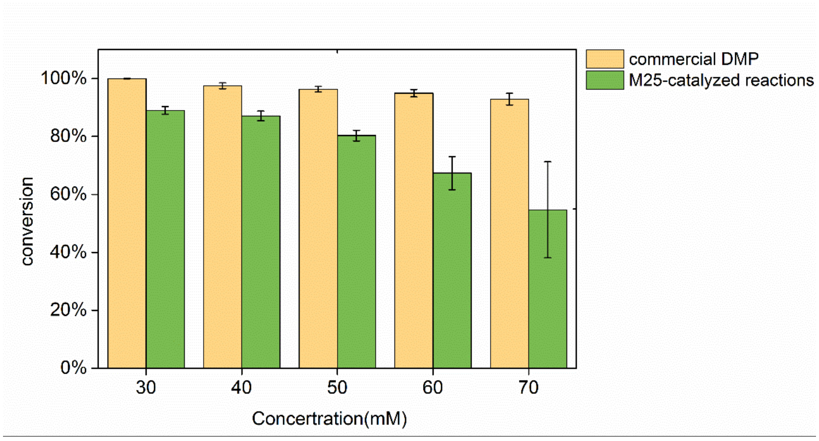

Supplementary Figure 3. Strain M38 catalyzed conversion of DMP obtained from commercial source and M25-catalyzed reactions.

d) Scale-up total biosynthesis of MPCA

Fed-batch fermentations with strain M11 were conducted with the protocols similar to that described above. After 28 h fermentation of M11, the concentration of L-threonine reached 35 g/L. Then the nutrient feeding solution delivering was stopped and the pH was adjusted to 8.0. The collected cells of strain M25 (12 g/L DCW) were added to the culture together with 2 mM NAD^+. When L-threonine was almost exhausted, the

collected cells of strain M38 (4.8 g/L DCW) were implemented to the culture. The pH was adjusted to 7.0 and the temperature was set at 30 ^{o}C for the biotransformation. The feeding of MIII nutrient was initiated and the glucose concentration was maintained below 1% (*w/v*). The concentrations of L-threonine, DMP, MPM and MPCA were monitored with HPLC.

References

[1] D. G. Gibson, L. Young, R. Y. Chuang, J. C. Venter, C. A. Hutchison, H. O. Smith. Nat Methods. 2009, 6, 343.

[2] Y. Jiang, B. Chen, C. Duan, B. Sun, J. Yang, S. Yang, Appl Environ Microbiol. 2015, 81, 2506.
